# Using CUSUM in real time to signal clinically relevant decreases in estimated glomerular filtration rate

**DOI:** 10.1186/s12882-022-02910-8

**Published:** 2022-08-18

**Authors:** Reyhaneh Zafarnejad, Steven Dumbauld, Diane Dumbauld, Mohammad Adibuzzaman, Paul Griffin, Edwin Rutsky

**Affiliations:** 1 Department of Industrial Engineering, Penn State University, 310 Leonhard Bldg., University Park, PA 16803 USA; 2 Regenstrief Center for Healthcare Engineering, Purdue University, West Lafayette, IN USA; 3 Good Samaritan Hospital FHC, Cincinnati, OH USA; 4 Department of Medical Informatics and Clinical Epidemiology, Oregon Health Sciences University, Portland, OR USA; 5 Division of Nephrology, University of Alabama at Birmingham, Birmingham, AL USA

**Keywords:** Chronic Kidney Disease (CKD), Early detection, CUSUM chart, Electronic Health Record (EHR), End Stage Kidney Disease (ESKD)

## Abstract

**Background:**

The electronic health record (EHR), utilized to apply statistical methodology, assists provider decision-making, including during the care of chronic kidney disease (CKD) patients. When estimated glomerular filtration (eGFR) decreases, the rate of that change adds meaning to a patient’s single eGFR and may represent severity of renal injury. Since the cumulative sum chart technique (CUSUM), often used in quality control and surveillance, continuously checks for change in a series of measurements, we selected this statistical tool to detect clinically relevant eGFR decreases and developed CUSUM_GFR_.

**Methods:**

In a retrospective analysis we applied an age adjusted CUSUM_GFR_, to signal identification of eventual ESKD patients prior to diagnosis date. When the patient signaled by reaching a specified threshold value, days from CUSUM signal date to ESKD diagnosis date (earliness days) were measured, along with the corresponding eGFR measurement at the signal.

**Results:**

Signaling occurred by CUSUM_GFR_ on average 791 days (se = 12 days) prior to ESKD diagnosis date with sensitivity = 0.897, specificity = 0.877, and accuracy = .878. Mean days prior to ESKD diagnosis were significantly greater in Black patients (905 days) and patients with hypertension (852 days), diabetes (940 days), cardiovascular disease (1027 days), and hypercholesterolemia (971 days). Sensitivity and specificity did not vary by sociodemographic and clinical risk factors.

**Conclusions:**

CUSUM_GFR_ correctly identified 30.6% of CKD patients destined for ESKD when eGFR was > 60 ml/min/1.73 m^2^ and signaled 12.3% of patients that did not go on to ESKD (though almost all went on to later-stage CKD). If utilized in an EHR, signaling patients could focus providers’ efforts to slow or prevent progression to later stage CKD and ESKD.

**Supplementary Information:**

The online version contains supplementary material available at 10.1186/s12882-022-02910-8.

## Background

Given the morbidity, mortality, and financial burden [1] of CKD, identifying eventual ESKD patients, when eGFR is ≥ 60 ml/min/1.73 m^2^, might provide opportunity to prevent deterioration leading to ESKD. Because of the silent nature of early kidney disease, and lack of recommendation by the US Preventive Services Task Force (USPSTF) for measuring serum creatinine in routine health screening [2], providers may not identify early CKD patients. The inverse relationship between serum creatinine (S_cr_) and eGFR results in underappreciation of early small increases in serum creatinine.

Rosansky suggested renal function trajectory might be more important than CKD staging [3]. The trajectory model measured in ml/min/1.73m^2^/year assumes a regression line fitted to data points over time. Determining trajectory is difficult as eGFR varies due to volume status, short term medication usage, underlying renal disease activity, age, and gender [4]. Time intervals between eGFR measurements in practice vary widely. Goodness-of-fit with regression analysis depends on observation number. Despite these limitations, Altman and Royston [4] emphasized the role time plays in a series of measurements. For the provider monitoring renal function, “one is specifically looking for the time when something changes.” Unfortunately, the pattern of renalfunction decline (as estimated by eGFR) can take several forms including linear, nonlinear, unidentifiable, and even positive [5]. This significantly limits the effectiveness of parametric approaches for identifying renal decline such as regression methods.

Using this concept that eGFR change rate is meaningful, CUSUM can be used for monitoring and detecting statistically significant change points in sequential data [6]. Often used for industrial process control, CUSUM provided a useful tool to analyze clinical data [7]. Subsequent CUSUM reviews demonstrated its use in healthcare applications [8–10]. Related to serial laboratory measurements, Peeks et al [11] identified changes in glucose levels using CUSUM. In nephrology, CUSUM was also used to determine initial dialysis stability [12] and transplant center quality [13].

By using a notification threshold value T, or signal, for a cumulative deviation over time from a given mean, a CUSUM chart can detect clinically relevant eGFR decreases in a patient’s series of measurements. The CUSUM statistic allows the assignment of weights (*w*) to each calculation, which tunes the signal for optimal sensitivity and specificity for detection of a future clinical risk outcome. In this retrospective data analysis using the statistic CUSUM_GFR_, ESKD diagnosis is the risk outcome, and tuned values of *w* and *T* optimize the performance of CUSUM_GFR._ Once the CUSUM_GFR_ value reaches threshold, the patient is likely to progress to ESKD.

Several researchers have estimated a natural decline in kidney function in healthy patients, and hence eGFR, with age. Cohen et al. estimate an annual decline in eGFR of 0.97 mL/min/1.73m^2^/year [14]. In a meta-analysis, Eriksen et al. estimate an annual decline in measured GFR of 0.72 mL/min/1.73m^2^/year [15]. The National Kidney Foundation report an annual decline in eGFR of 0.81 mL/min/1.73m^2^/year [16]. The CUSUM statistic can be easily modified to account for this natural progression.

## Methods

**Fig. 1:**
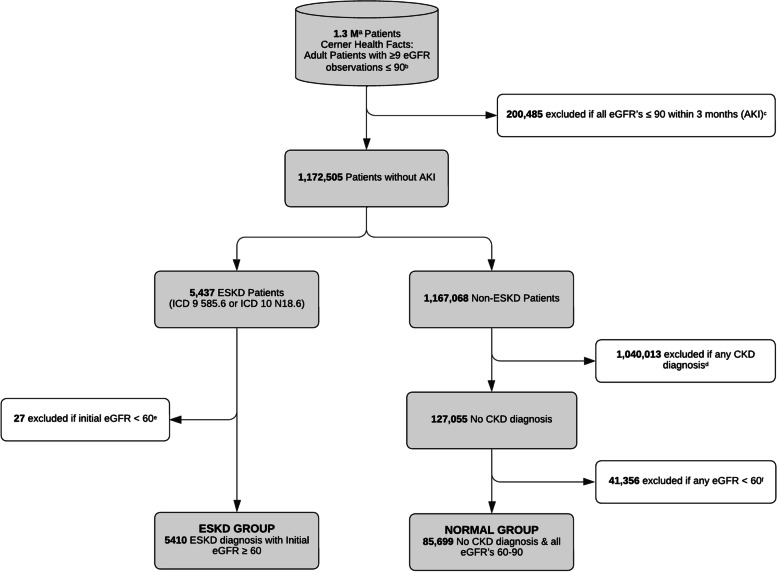
Selection criteria. **a** Million. **b** All eGFRs in min/ml/1.73m^2^. **c** Acute kidney injury. **d** Patients excluded for any ICD9/10 CKD diagnosis (see Appendix Table 1). **e** Excluded any ESKD Group patient with initial eGFR measurement < 60 min/ml/1.73m^2^. **f** Excluded any Normal Group patient with any eGFR < 60 min/ml/1.73 m.^2^

We selected participants from Cerner Health Facts database (Fig. 1), containing EHR data of 1.3 million adult patients with multiple S_cr_measures from 2010—2019. We calculated eGFRs using the 2021 CKD-EPI Eq [17]. for all patients. Patients with acute kidney injury (all eGFR’s < 90 ml/min/1.73m^2^ within 3 months) were excluded, and the remaining were divided into two mutually exclusive subgroups (Normal and ESKD groups) based on ICD9/10 diagnosis: a group diagnosed with ESKD (ICD9 585.6 or ICD10 N18.6) as the outcome, and a group without ESKD. This allows for the estimation of sensitivity and specificity of the method. To determine intrinsic, non-pathologic variation in eGFR in the non-ESKD patients, we excluded patients with any CKD Diagnoses (Appendix Table 1), and those with any eGFR measurement < 60. This Normal Group totaled 85,699 patients and were used to calculate the eGFR mean, $\widehat{\mu }$, and standard deviation, $\widehat{\sigma }$, for use in the CUSUM_GFR_ statistic and were included in CUSUM_GFR_ calculations. To signal ESKD patients as early as possible, we excluded patients in the ESKD Group with initial eGFR < 60 mL/min/1.73m^2^ (5,410 patients). LOINC codes (Appendix Table 2) were used to collect laboratory data including S_cr_ in all patients.

We use the following cumulative statistic:

$${\mathrm{CUSUM}}_{\mathrm{GFRi}}=min\left[0,\left(\frac{eGFRi-\widehat{\mu_i}}{\widehat\sigma}\right)+w+CUSUM_{GFRi}-1\right]$$

where *CUSUM*_*GFR0*_ = 0, $\widehat{{\mu }_{i}}$ is the mean of eGFR and $\widehat{\sigma }$ is the standard deviation for patients in the Normal Group, and *eGFR*_*i*_ is the *i*th measurement of eGFR for each patient in both groups. Note CUSUM_GFR_ will always be less than or equal to zero due to the use of minimum operator, which ensures that CUSUM_GFR_ only detects significant decline in eGFR. If the CUSUM_GFR_ calculation falls below the threshold signal value *T*, the patient signals likelihood of progressing to ESKD.

Given a natural decline in healthy patients of 0.81 mL/min/1.73m^2^/year [16], the age adjusted mean of the normal group $\widehat{{\mu }_{i}}$ is determined as follows:

$$\widehat{{\mu }_{i}}= \widehat{{\mu }_{0}}-0.81\Delta t$$

where $\widehat{{\mu }_{0}}$ is the mean eGFR value for the normal group at the age of the patient during their first reported eGFR measurement and $\Delta t$ is the different in years between the age of the patient at measurement *I* and their first measurement.

**Fig. 2:**
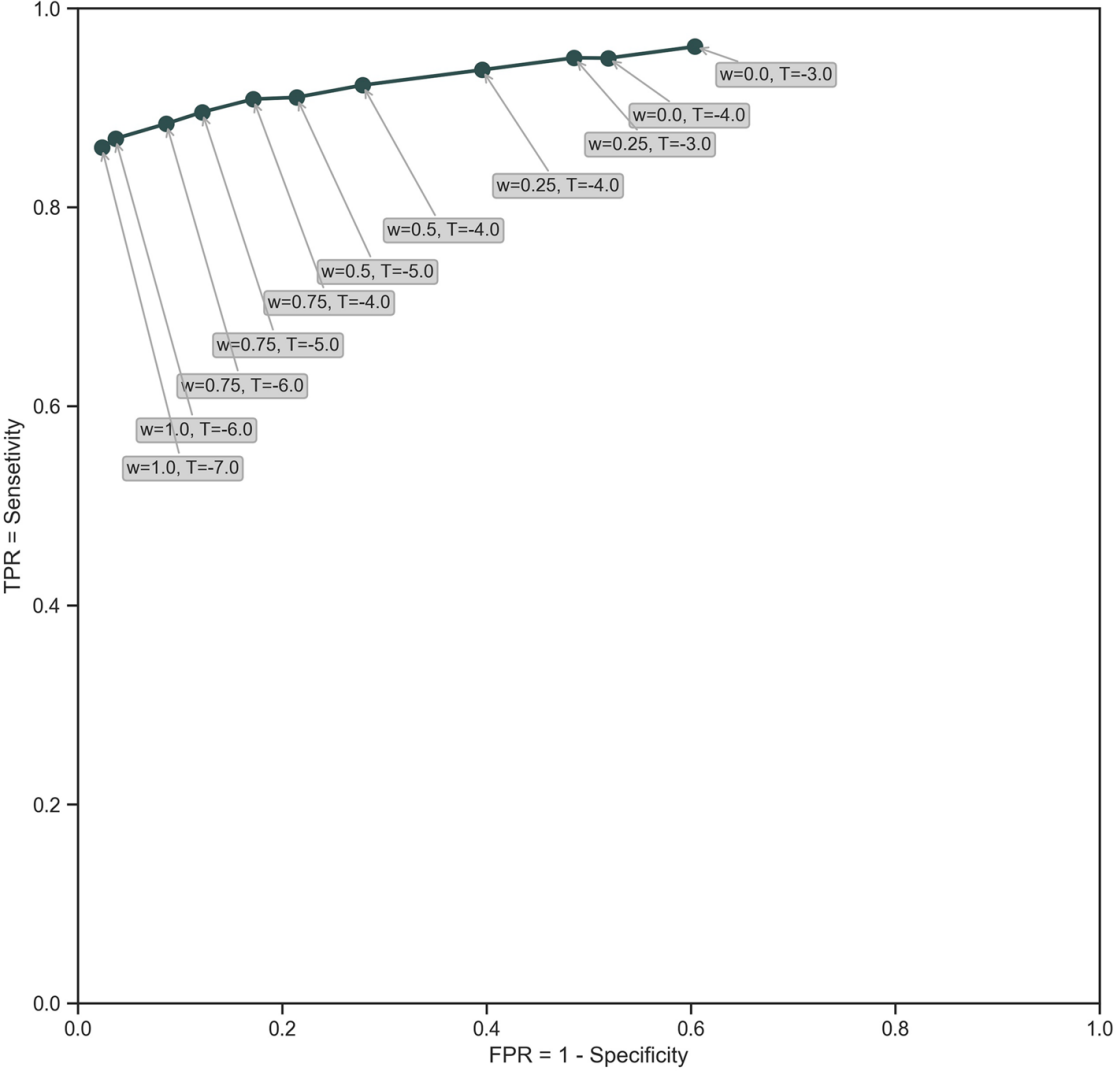
Receiver Operating Characteristic (ROC) curve with sample values for w (tuning parameter) and T (signal value) demonstrating the effect on performance measures (sensitivity and specificity)

The parameters *w* and *T* are chosen to balance the tradeoff between false positive and false negative outcomes. The parameter, *w*, is a tuning parameter that is an allowable, clinically meaningful, shift in the cumulative measurement, determined as noted below. To determine the best choices for *T* and *w*, we analyzed the Normal and ESKD Groups using k-fold cross validation (k = 10) for several *w* and *T* values. A receiver operator characteristics (ROC) curve (Fig. 2) revealed the best sensitivity, specificity, and accuracy for T, the threshold signal value. When signaled, the patient’s eGFR and days prior to ESKD diagnosis were recorded. The difference between signal date and ESKD diagnosis date defines earliness. We determined total population performance measures and when stratified by the sociodemographic variables of age, sex, and race and the clinical factors of hypertension, diabetes, cardiovascular disease, and hypercholesterolemia.

## Results

Baseline data on demographics, diagnoses, laboratory results, and medications for the Normal and ESKD Groups are provided in Table 1. The ESKD Group had a significantly higher proportion that were male, Black, Native American, Asian/Pacific Islander, and Hispanic, and higher rates of smoking, hypertension, diabetes, cardiovascular disease, and history of cancer, hypercholesterolemia, and urinary tract abnormalities. All measured laboratory results were significantly different between the Normal Group and the ESKD Group. The ESKD Group had higher rates of non-steroidal anti-inflammatory drug, proton pump inhibitor, and lithium use.

**Table 1:** Baseline demographics, diagnoses, laboratory results, and medications data for normal and ESKD patient groups

	**Normal Group** (*n* = 85,699)	**ESKD Group** (*n* = 5,410)
**DEMOGRAPHICS**		
Mean Age in years**	64.5	57.9
Sex*		
Number Female (percent)	46,456 (54%)	2,354 (44%)
Number Male (percent)	39,182 (46%)	3,056 (56%)
Race/Ethnicity*		
Number Black (percent)	5,826 (7%)	1,147 (21%)
Number Native American (percent)	181 (0%)	110 (2%)
Number Asian/Pacific Islander (percent)	1,062 (1%)	114 (2%)
Number Hispanic (percent)	26 (0%)	55 (1%)
Number Middle Eastern/Indian (percent)	490 (1%)	7 (0%)
Number White (percent)	69,294 (81%)	3,589 (67%)
Number Biracial (percent)	45 (0%)	7 (0%)
Number Unknown (percent)	8,754 (10%)	381 (7%)
Number with History of Smoking (percent)*	15,063 (18%)	2,423 (45%)
**DIAGNOSES**		
Number with Hypertension (percent)*	46,502 (54%)	4,816 (89%)
Number with Diabetes Mellitus (percent)*	22,215 (26%)	3,403 (63%)
Number with Cardiovascular Disease (percent)*		
Coronary Artery Disease	12,812 (15%)	2,346 (43%)
Cerebrovascular Disease (CVA, Stroke)	5,041 (6%)	764 (14%)
Peripheral Vascular Disease	4,338 (5%)	1,168 (22%)
Number with History of Cancer (percent)*	10,294 (12%)	767 (14%)
Number with Hypercholesterolemia (percent)*	48,716 (57%)	3,404 (63%)
Number with History of Urinary Tract Abnormalities (percent)*	4633 (5%)	1512 (28%)
**LABORATORY RESULTS**		
Urine Microalbumin/Creatinine (mg/g)*		
Number patients < 30 (percent)	12,755 (81.7%)	31 (25.6%)
Number patients between 30 and 300 (percent)	2,593 (16.6%)	40 (33.1%)
Number of patients > = 300 (percent)	255 (1.7%)	50 (41.3%)
Urine Protein/Creatinine (g/g) (se)*	0.11 (0.010)	3.91 (1.247)
Hemoglobin A1c (g/dL) (se)*	5.3 (0.008)	7.2 (0.062)
Hemoglobin (g/dL) (se)*	13.4 (0.002)	10.9 (0.037)
Serum Calcium (mg/dL) (se)*	9.4 (0.001)	8.8 (0.014)
Serum Cholesterol (mg/dL) (se)*	182 (0.051)	159 (1.814)
Serum Albumin (g/dL) (se)*	4.1 (0.001)	3.2 (0.013)
Serum Phosphorus (mg/dL) (se)*	3.4 (0.001)	4.2 (0.034)
Number of patients Hepatitis C positive (percent)*	945 (1%)	237 (4%)
**MEDICATION**		
Number with any NSAID Use (ibuprofen, naproxen, etc.) (percent)*	16,459 (19%)	2,518 (47%)
Number with any Proton Pump Inhibitor Use (omeprazole, etc.) (percent)*	9,689 (11%)	4,031 (75%)
Number with Bipolar Drug Use (Lithium) (percent)*	233 (0%)	28 (1%)

*se* Standard error

^*^ Significant difference in means between normal and ESKD Groups based on chi-squared test (*p* < 0.05)

^**^ Significant difference in means between normal and ESKD Groups based on *t*-test (*p* < 0.05)

The overall mean eGFR value for the Normal Group was 85.07 mL/min/1.73 m^2^ (se = 0.03). Mean eGFR values for the Normal Group by age are shown in Appendix Table 3. Using Kolmogorov Smirnov goodness of fit test, we could not reject the hypothesis that the mean eGFR for the Normal Group was normally distributed ($\alpha$=0.05). The values *CUSUM*_*GFR0*_ = 0, *w* = 0.75, and *T* = –4.0, gave best mean accuracy (0.878), mean sensitivity (0.897), and mean specificity (0.877) to signal a patient likely to progress to ESKD. Note that w = 0.75 corresponds to a meaningful cumulative eGFR shift of 0.75 $\widehat{\sigma }$ = 5.84 mL/min/1.73m^2^. Those patients who signaled in the Normal Group were considered false positives, and those in the ESKD Group who failed to signal false negatives.

**Fig. 3:**
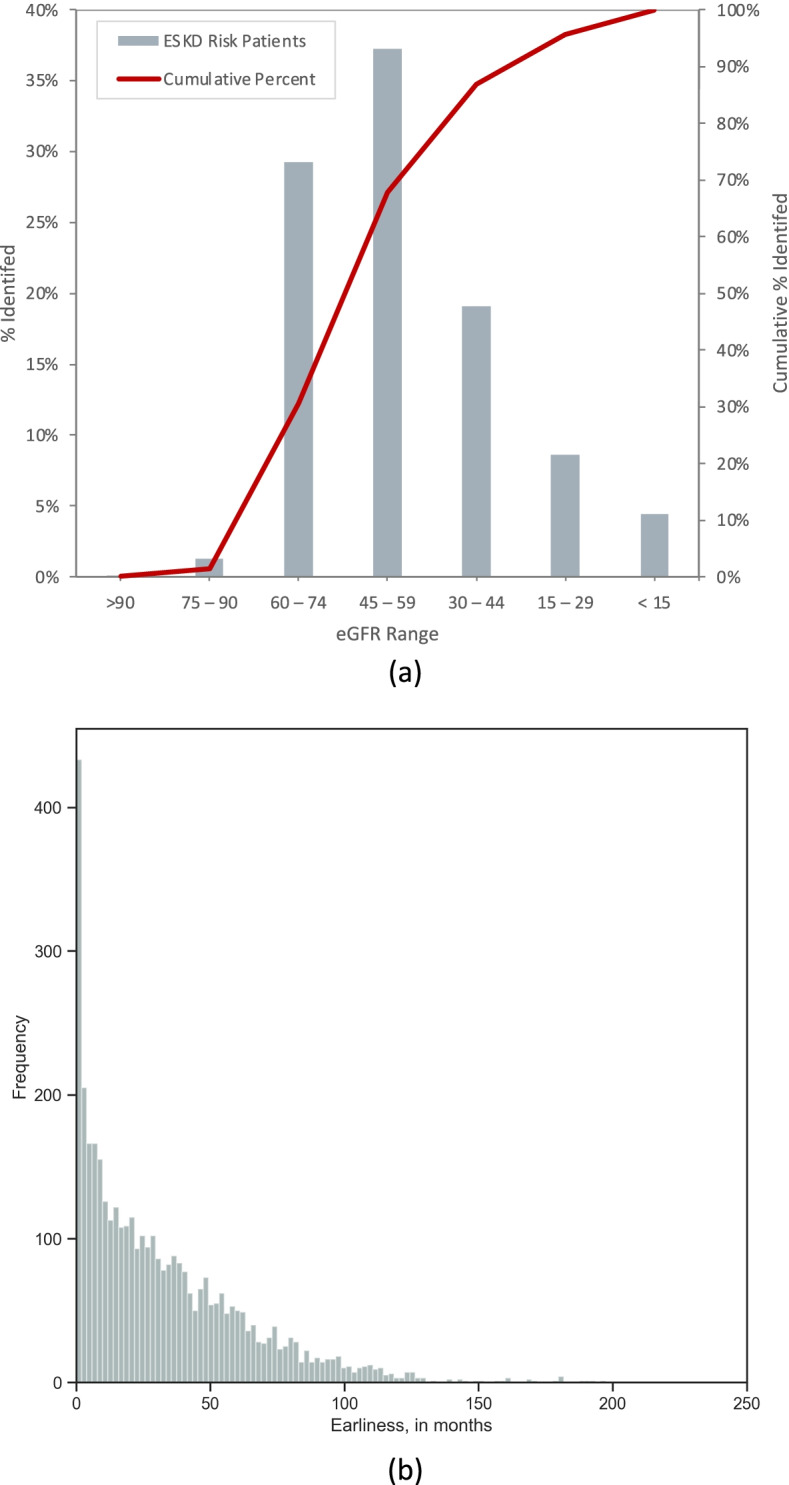
eGFR at CUSUM_GFR_ Signal, in ml/min/1.73m2/year (**a**); earliness (in months) from CUSUM_GFR_ Signal (CUSUM_GFRi_ <  = –4.0) to ESKD diagnosis. Mean earliness is 26.3 months. Only those patients correctly identified prior to their diagnosis were included (**b**)

Figure 3 shows the distribution of eGFR at time of risk signal, and the distribution of signal earliness to actual diagnosis date. Of those in the ESKD Group who signaled as likely to progress, 86.9% did so when eGFR was $\ge$ 30, 67.9% when $\ge$ 45, and 30.6% when $\ge$ 60 mL/min/1.73m^2^ and signaled 791 days (mean earliness) prior to ESKD diagnosis date (median earliness 361 days). Also note that 12.3% of patients that signaled as likely to progress to ESKD do not do so, however, almost all of these went on to later stage CKD (CKD level 4 and 5), which would still benefit from early intervention.

**Fig. 4:**
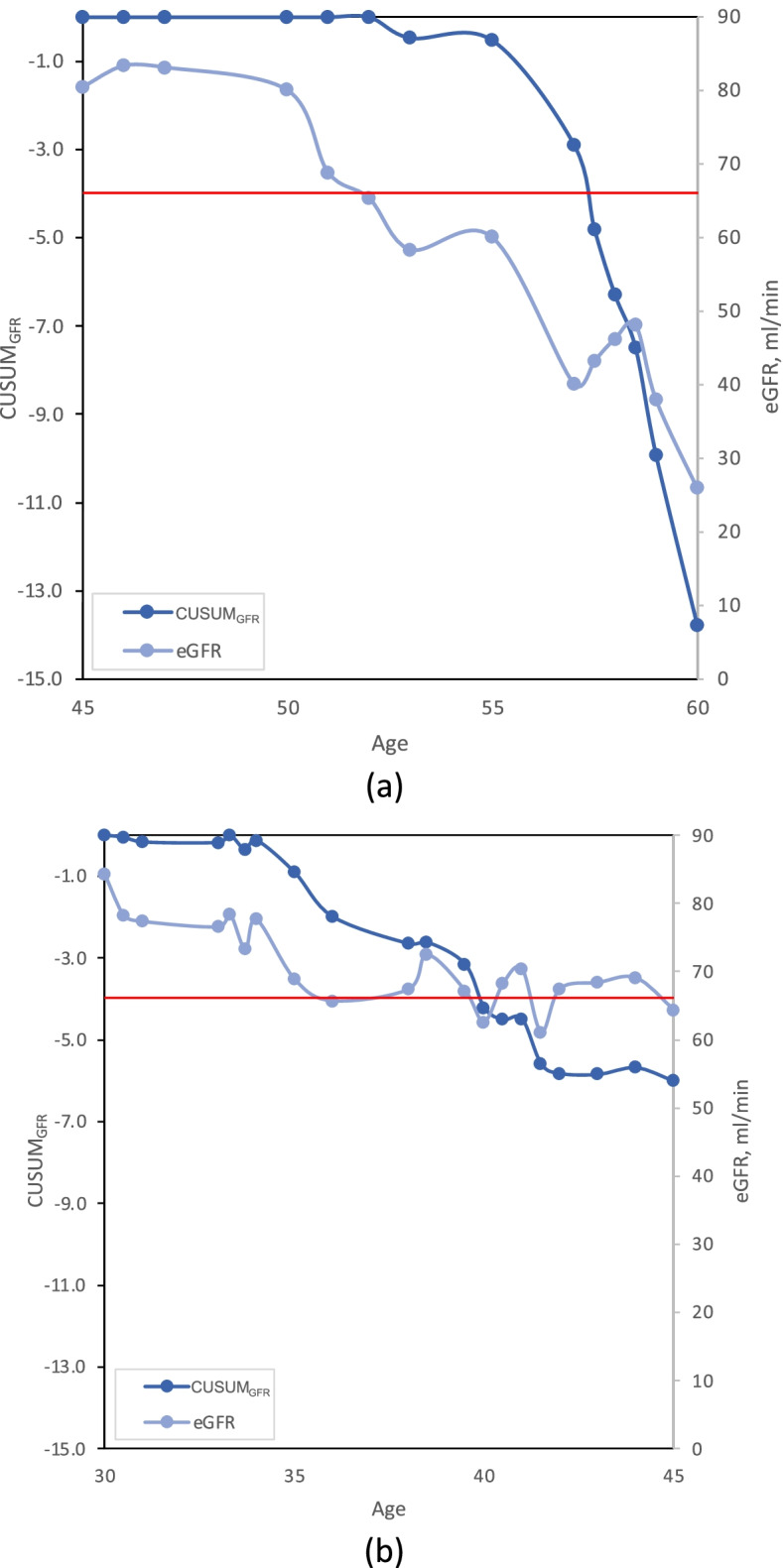
Two examples of CUSUM_GFR_ for patients that went on to ESKDillustrating a rapid decrease (**a**) and graduate decrease (**b**). Both patients were identified as at risk at the observation falling below –4.0

CUSUM_GFR_ signal in two ESKD patients is illustrated in Fig. 4. The first patient had a rapid decline in eGFR starting at age 57 are fell below 30 mL/min/1.73m^2^ at age 60. The signal occurred soon after the initial drop at age 57, three years before diagnosis. The second patient had a slow decline in eGFR, and never fell below 60 mL/min/1.73m^2^ before the age of 45. However, they were correctly signaled to be at risk for ESKD at age 40, well before their diagnosis at age 56.

CUSUM_GFR_ performance is shown in Appendix Table 4 for population subgroups based on sociodemographic factors and clinical risk conditions. Accuracy, sensitivity, and specificity did not vary significantly by subgroup compared to the total values, except in two subgroups. Sensitivity dropped for the non-hypertension subgroup and specificity dropped for the adults over 65 years of age. Mean earliness was greater for patients with cardiovascular disease (1027 days), hypercholesterolemia (971 days), diabetes (940 days), and hypertension (852 days). Black patients signaled earlier than non-Black patients (905 versus 759 days respectively).

## Discussion

Global prevalence of CKD was 9.1% in 2017 and has increased by over 29% since 1990 [18]. CKD progression to ESKD affected over 746,557 individuals in the US in 2017 and is projected at 1.2 M by 2030 [19]. ESKD is a leading cost in healthcare with Medicare spending for ESKD totaling $35.9B in 2017, 7.2% of Medicare paid claims [2]. Earlier identification of CKD patients likely to progress might reduce the incidence of ESKD.

Despite previous studies using various models [20–25] to predict CKD progression, identification of these at-risk patients is challenging. In early renal injury, S_cr_increases are subtle, with small increments representing substantial reductions in eGFR, and may be unrecognized. While normal individuals show a fairly constant rate change over a lifetime [26], CKD patients do not have predictable patterns of progression [5]. We include several typical examples of eGFR change over time for patients who developed ESKD in our data in Appendix Fig. 1, and the change over time varies considerably. In the absence of parametric patterns, linear regression analysis does not perform reliably, and any non-pathologic eGFR change measurement must be differentiated from pathologic causes. No widely accepted method for computing eGFR changes for individual patients is available and CUSUM_GFR_ provides a useful computed statistical application easily incorporated within any healthcare system’s EHR.

In our retrospective data analysis using CUSUM_GFR_, it is possible to signal CKD patients likely to progress early in the course of their renal disease. We emphasize that this statistic provides continual monitoring, looking for significant change in eGFR for every serum creatinine measurement for every patient enrolled in a healthcare system’s EHR. With the current eGFR indication for nephrology CKD consultation commonly accepted at < 30 mL/min/1.73m^2^, opportunity for best intervention at higher eGFR levels may be lost. Since over a quarter of ESKD Group patients signaled likeliness to progress when eGFR ≥ 60, this indication should be reconsidered. Inclusion of CUSUM_GFR_ within the EHR fits directly into provider workflow since the signal alert is to the provider only when *T*exceeds the threshold value and would lead the provider to evaluation and treatment algorithms. Early recognition of the CKD patients who signal early might reduce ESKD incidence, and decrease the high morbidity and mortality associated with late nephrology referral [27, 28].

We found that patients with clinical risk factors (cardiovascular disease, diabetes, hypertension, and hypercholesterolemia) had a greater mean earliness signal compared to those with no risk factors. Black patients, similarly, had a greater mean earliness signal as well. This could be due to the Black patients in our study having a higher rate of co-morbidities (clinical risk factors) compared to non-Black patients (data not shown).

There are several limitations to our study. First, it is not reported in the Cerner data which assay type (e.g., Jaffe or enzymatic) was used for the SCr measurements, and this likely varied by lab. It is possible that differences in assay type could lead to different results. Second, although we used a large patient population in our study, it was not a random sample and may not be nationally representative. Therefore, there could be bias in the estimated parameters. Finally, our selection criteria for the Normal Group required a patient to have at least nine eGFR measurements in the EHR. This implies that the patients were regular utilizers of healthcare and hence may be at higher risk than “normal” patients nationally. It further implies that application of the method for a particular provider may require retrospective data analysis on their specific population to estimate mean eGFR by age and standard deviation for their “normal” population.

Retrospective analysis of CUSUM_GFR_ in other medical databases is needed to validate these findings, but ultimately the benefit of CUSUM_GFR_ can only be truly estimated through randomized prospective studies. Such prospective studies could determine if early detection of risk and implementation of interventions could reduce the decline in kidney function and incidence of ESKD.

Beyond signaling providers of CKD patients likely to progress to ESKD, other CUSUM_GFR_ applications include timing referral for transplantation and placement of arteriovenous fistulae, correlating CUSUM_GFR_ signaling with renal biopsy activity staging, and has potential use as an endpoint in randomized controlled trials. Not intended as a stand-alone statistic in the care of CKD patients, CUSUM_GFR_ can serve as an important new tool for primary care provider and nephrologist alike.

## Supplementary Information

**Additional file 1: Appendix Table 1.** ICD 9 and ICD 10 codes used in the analysis. The asterisks correspond to wildcard values. ICD 9 and ICD 10 codes in bold were used in selection criteria. **Appendix Table 2.** LOINC codes used in the analysis. **Appendix Table 3.** Mean eFGR values for the Normal Group by age. **Appendix Table 4.** Performance measures (accuracy, sensitivity, specificity, mean earliness, median earliness) for CUSUM_GFR_ based on population subgroups. **Appendix Figure 1.** Examples of eGFR changes in patients that went on to ESKD.

## Data Availability

The data that support the findings of this study are not publicly available. They were made available the research team through a data use agreement with Cerner. https://www.cerner.com/ap/en/solutions/data-research. The source code for the analysis may be found at: https://github.com/Rey-Zafarnejad/Identifying_Clinically_Relevant_Decrease_in_Estimated_Glomerular_Filtration_Rate.

## Abbreviations

CKD: Chronic kidney disease
CUSUM: Cumulative sum statistic
eGFR: Estimated glomerular filtration rate
HER: Electronic health record
ESKD: End stage kidney disease
m: Meters
ml: Milliliters
Scr: Serum creatinine
se: Standard error
