# Supplementary material for: Using CUSUM in real time to signal clinically relevant decreases in estimated glomerular filtration rate
Source: BMC Nephrol. 2022 Aug 18;23:287. doi: 10.1186/s12882-022-02910-8 (PMC9389810; doi:10.1186/s12882-022-02910-8)

**Appendix Table 1**. ICD 9 and ICD 10 codes used in the analysis. The asterisks correspond to wildcard values. ICD 9 and ICD 10 codes in bold were used in selection criteria.

| **DIAGNOSIS** | **ICD10** | **ICD9** |
| --- | --- | --- |
| **Chronic Kidney Disease (CKD)** |  |  |
| **Chronic kidney disease, Stage I** | **N18.1** | **585.1** |
| **Chronic kidney disease, Stage II (mild)** | **N18.2** | **585.2** |
| **Chronic kidney disease, Stage III (moderate)** | **N18.3** | **585.3** |
| **Chronic kidney disease, Stage IV (severe)** | **N18.4** | **585.4** |
| **Chronic kidney disease, Stage V** | **N18.5** | **585.5** |
| **End stage kidney disease** | **N18.6** | **585.6** |
| **Chronic kidney disease, unspecified** | **N18.9** | **585.19** |
| **Unspecified kidney failure** | **N19** | **586** |
| **Other disorders of kidney and ureter** | **N25-N29** | **581.*, 583.*, 586.*, 587.*, 588.*** |
| **Glomerular diseases** | [**N00-N08**](about:blank) | **580.*, 582.*** |
| **Acute kidney failure** | [**N17**](about:blank) | **584.*** |
| **Kidney replaced by transplant** | **V42.0** | **Z94.0** |
| Nicotine/Tobacco Dependency |  |  |
| Personal History of tobacco use | - | V15.82 |
| Personal History of nicotine dependence | Z87.891 | - |
| Tobacco dependence | - | 305.1 |
| Nicotine dependence | F17.*** | - |
| Tobacco use NOS | Z72.0 | - |
| Tobacco use disorder complicating pregnancy | O99.33* | 649.0* |
| Hypertension, All Types |  |  |
| Essential HTN | I10 | 401.0, 401.1, 401.9 |
| Hypertensive heart disease | I11.* | 402.** |
| Hypertensive chronic kidney disease | I12.* | 403.** |
| Hypertensive heart and chronic kidney disease | I13.** | 404.9*, 404.0*, 404.1** |
| Secondary HTN | I15.* | 405.** |
| Hypertensive Crisis | I16.* | - |
| Pre-existing HTN complicating pregnancy | O10.*** | 642.11, 642.14 |
| Pre-existing HTN with pre-eclampsia | O11.* | 642.7* |
| Diabetes Mellitus |  |  |
| Diabetes mellitus due to underlying condition | E08.**** | 249.** |
| Drug or chemical induced diabetes mellitus | E09.**** | - |
| Type 1 diabetes mellitus | E10.**** | - |
| Type 2 diabetes mellitus | E11.**** | - |
| Other specified diabetes mellitus | E13.**** | - |
| Pre-existing Diabetes mellitus type 1 in pregnancy | O24.0** | - |
| Pre-existing Diabetes mellitus type 2 in pregnancy | O24.1** | - |
| Unspecified Pre-existing Diabetes mellitus in pregnancy | O24.3** | - |
| All diabetes mellitus diagnoses in ICD9 |  | 249.**, 250.** |
| Cardiovascular Disease/Coronary Artery Disease |  |  |
| Atherosclerotic disease of native coronary artery | I25.1** | 414.01, 414.03, 414.8, 414.9, 414.3, 414.4 |
| Old Myocardial Infarction | I25.2 | 412 |
| Ischemic Cardiomyopathy | I25.5 | 414.8 |
| Silent myocardial ischemia | I25.6 | 414.8 |
| Atherosclerosis of coronary artery bypass graft and in Heart transplant | I25.7*** | 414.02, 414.06, 414.05, 414.04, 414.00 |
| Other forms of chronic ischemic heart disease | 125.8** | 414.8 |
| Chronic ischemic heart disease unspecified | 125.9 | 414.9 |
| Cardiovascular Disease/Cerebrovascular Disease |  |  |
| Cerebral Infarction due to thrombosis due to pre-cerebral arteries | I63.0** | 433.** |
| Cerebral Infarction due to unspecified occlusion pre-cerebral arteries | I63.2** | - |
| Cerebral Infarction due to thrombosis of cerebral artery | I63.3** | 434.0* |
| Cerebral Infarction due to unspecified occlusion cerebral arteries | I63.5** | 434.9 |
| Other cerebral infarction | I63.8* | - |
| Cerebral infarction unspecified | I63.9 | - |
| Occlusion and stenosis of pre-cerebral arteries | I65.** | 433.** |
| Occlusion and stenosis of cerebral arteries | I66.** | 434 |
| Cerebral atherosclerosis | I67.2 | 434.9* |
| Hypertensive encephalopathy | I67.4 | 437.2 |
| Moyamoya disease | I67.5 | 437.5 |
| Other specified cerebrovascular diseases | I67.8*** | 436 |
| Cerebrovascular diseases unspecified | I67.9 | 437.9 |
| Cerebral atherosclerosis for ICD9 codes |  | 437.0, 437.1, 437.8 |
| Cardiovascular Disease/Peripheral Vascular Disease |  |  |
| Atherosclerosis | I70.*** | 440.**, 443.8*, 443.9 |
| Sickle Cell Disorders | D57.*** | 282.6* |
| Personal history of malignant neoplasm | Z85.*** | V10.** |
| Disorders of Lipoprotein Metabolism | E78.** | 272.* |
| History of urinary tract abnormalities |  |  |
| Obstructive and Reflux Uropathy | N13.*** | 591, 593.3, 593.7*, 593.89, 593.9, 599.6, 599.8, 599.9 |
| Unspecified contracted kidney | N26.* | 589.9 |
| Small kidney unknown cause | N27.* | 589.* |
| Other disorders of kidney and ureter, NEC | N28.** | - |
| Neuromuscular dysfunction of bladder NEC | N31.* | 596.5* |
| Bladder neck obstruction | N32.0 | 596 |
| Childhood kidney disease | Any N00-N99 with onset prior to age 18 | Any 580-599 with onset prior to age 18 |
| Medications (better to search for drugs specifically than the ICD10 code) |  |  |
| Long Term Use of NSAID's | Z79.1 | V58.64 |
| Other Long Term Drug Therapy | Z79.899 |  |

**Appendix Table 2**. LOINC codes used in the analysis.

| **LOINC Codes** | **UOM** |
| --- | --- |
| Urine Albumin/Creatinine Ratio |  |
| LOINC: 32294-1 | mg/mmol |
| LOINC: 13705-9 | mg/g creat |
| LOINC: 14585-4 | mmol/mol{creat} |
| LOINC: 32294-1 | mg/mmol |
| LOINC: 89998-9 | mg/mol{creat} |
| LOINC: 9318-7 | mcg/mg cr |
| Urine Protein/Creatinine Ratio -> mg/dL |  |
| LOINC: 34366-5 | g/mmol{creat} |
| LOINC: 13801-6 | mg/g{creat} |
| LOINC: 2890-2 | mg/g{creat} |
| LOINC: 40486-3 | gm/mmol_cre |
| Urine Microalbumin/Creatinine Ratio |  |
| LOINC: 14959-1 | mg/gm cr |
| LOINC: 30000-4 | mg/mmol{creat} |
| LOINC: 58447-4 | mg/g{creat} |
| LOINC: 77253-3 | mg/mmol{creat} |
| LOINC: 89998-9 | mg/mol{creat} |
| 24 hr Urine Protein |  |
| LOINC: 2889-4 | mg/24Hr (also listed as g/24Hr) |
| LOINC: 21482-5 | mg/dL |
| LOINC: 3167-4 |  |
| LOINC: 3168-2 |  |
| Hemoglobin A1c |  |
| LOINC: 59261-8 | % or mmol/mol |
| LOINC: 71875-9 | Reported as decimal fraction |
| LOINC: 41995-2 | g/dL |
| LOINC: 17856-6 | % Hgb |
| LOINC: 62388-4 | % |
| LOINC: 4548-4 | % Hgb |
| Hemoglobin |  |
| LOINC: 718-7 | g/dL |
| Serum Calcium |  |
| LOINC: 17861-6 | mg/dL |
| LOINC: 2000-8 | mEq/L or mmol/L (2 mEq/mmol) |
| Serum Bicarbonate |  |
| LOINC: 1963-8 | mmol/L |
| LOINC: 1959-6 | mEq/L |
| LOINC: 1962-0 | mmol/L |
| LOINC: 14627-4 | mmol/L |
| HIV Positive |  |
| LOINC: 68961-2 | ORD |
| LOINC: 89374-3 | ORD |
| LOINC: 86233-4 | ORD |
| LOINC: 85686-4 | ORD |
| LOINC: 7917-8 | ORD |
| Hepatitis C Positive |  |
| LOINC: 72376-7 | ORD |
| LOINC: 22327-1 | Qn result |
| LOINC: 5198-7 | Qn result |
| LOINC: 16128-1 | ORD |
| LOINC: 13955-0 | ORD |
| Serum Cholesterol |  |
| LOINC: 2093-3 | mg/dL |
| LOINC: 48620-9 | mg/dL |
| LOINC: 14647-2 | mmol/L |
| Serum Albumin |  |
| LOINC: 77148-5 Method BCP | g/L |
| LOINC: 61152-5 Method BCP | g/dL |
| LOINC: 62234-0 Method BCP | mmol/L |
| LOINC: 61151-7 Method BCG | g/L |
| LOINC: 62235-7 Method BCG | mmol/L |
| LOINC: 1751-7 Unknown Method | g/L or mg/dL |
| LOINC: 54347-0 Unknown Method | mmol/L |
| Serum Phosphorus |  |
| LOINC: 2777-1 | mg/dL |
| LOINC: 14879-1 | mmol/L |

**Appendix Table 3**. Mean eFGR values for the Normal Group by age.

| **Age Range** | **Sample Size** | **Mean eGFR in Normal Group** |
| --- | --- | --- |
| 18-25 | 88 | 88.634 |
| 25-30 | 240 | 91.039 |
| 30-35 | 509 | 89.386 |
| 35-40 | 912 | 87.040 |
| 40-45 | 2090 | 86.878 |
| 45-50 | 4884 | 87.681 |
| 50-55 | 8030 | 86.390 |
| 55-60 | 9966 | 84.543 |
| 60-65 | 10102 | 84.310 |
| 65-70 | 12411 | 84.752 |
| 70-75 | 12451 | 85.031 |
| 75-80 | 9433 | 84.499 |
| 80-85 | 5394 | 83.953 |
| - 85 | 3363 | 82.954 |

**Appendix Table 4**. Performance measures (accuracy, sensitivity, specificity, mean earliness, median earliness) for CUSUM_GFR_ based on population subgroups.

| **Sub group** | **N (ESKD)** | **N (Normal Group)** | **Accuracy** | **Sensitivity** | **Specificity** | **Mean earliness (ESRD diagnosis)** | **SE of earliness (ESRD diagnosis)** |
| --- | --- | --- | --- | --- | --- | --- | --- |
| **Adults under 65** | 3614 | 41067 | 0.846 | 0.929 | 0.839 | 798 days | 15 days |
| **Adults above 65** | 1796 | 44632 | 0.909 | 0.834 | 0.912 | 777 days | 24 days |
| **Female** | 2354 | 46456 | 0.871 | 0.894 | 0.870 | 825 days | 20 days |
| **Male** | 3056 | 39182 | 0.887 | 0.900 | 0.886 | 765 days | 16 days |
| **Black** | 1147 | 5826 | 0.845 | 0.928 | 0.829 | 905 days* | 29 days |
| **Other (Non-Black)** | 4263 | 79873 | 0.881 | 0.889 | 0.881 | 759 days | 14 days |
| **Hypertension** | 4816 | 46502 | 0.887 | 0.903 | 0.886 | 852 days* | 14 days |
| **Non Hypertension** | 594 | 39197 | 0.866 | 0.847 | 0.867 | 271 days | 24 days |
| **Diabetes** | 3403 | 22215 | 0.893 | 0.919 | 0.889 | 940 days* | 16 days |
| **Non Diabetes** | 2007 | 63484 | 0.873 | 0.860 | 0.873 | 522 days | 19 days |
| **Cardiovascular Disease** | 2895 | 17671 | 0.904 | 0.920 | 0.901 | 1027 days* | 18 days |
| **Non Cardiovascular Disease** | 2515 | 68028 | 0.871 | 0.882 | 0.871 | 568 days | 17 days |
| **Hypercholesterolemia** | 3404 | 48716 | 0.884 | 0.904 | 0.883 | 971 days* | 17 days |
| **Non Hypercholesterolemia** | 2006 | 36983 | 0.870 | 0.885 | 0.869 | 480 days | 17 days |
| **Total** | **5410** | **85699** | **0.878** | **0.897** | **0.877** | **791 days** | **12 days** |

* Significant difference in mean detection earliness between subgroup pair based on *t*-test (p < 0.05).

**Appendix Figure 1**. Examples of eGFR changes in patients that went on to ESKD.


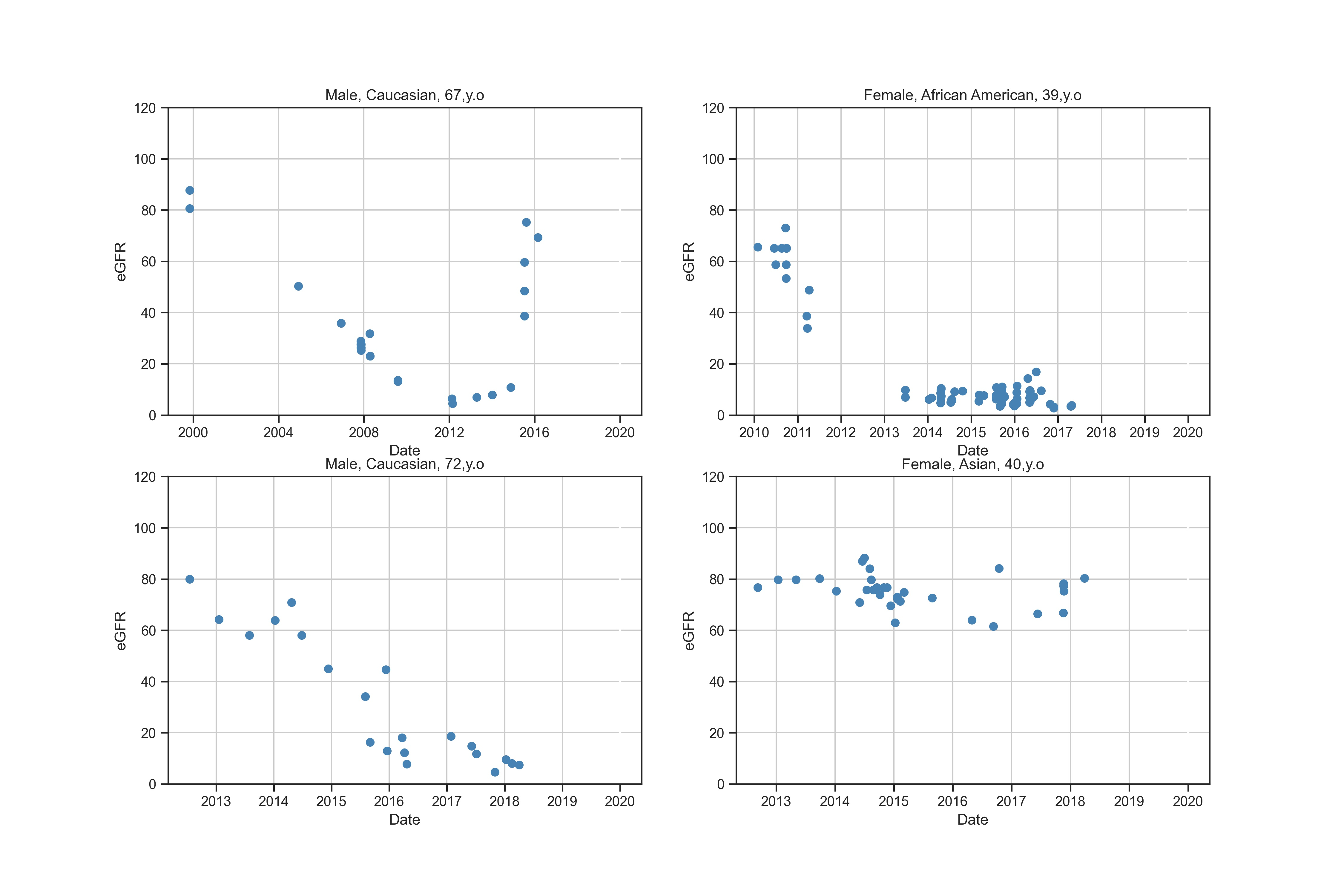

Supplement: Supplementary file 1 — Additional file 1: Appendix Table 1. ICD 9 and ICD 10 codes used in the analysis. The asterisks correspond to wildcard values. ICD 9 and ICD 10 codes in bold were used in selection criteria. Appendix Table 2. LOINC codes used in the analysis. Appendix Table 3. Mean eFGR values for the Normal Group by age. Appendix Table 4. Performance measures (accuracy, sensitivity, specificity, mean earliness, median earliness) for CUSUMGFR based on population subgroups. Appendix Figure 1. Examples of eGFR changes in patients that went on to ESKD. [file 12882_2022_2910_MOESM1_ESM.docx]
